# Association of Virulence and Antibiotic Resistance in *Salmonella*—Statistical and Computational Insights into a Selected Set of Clinical Isolates

**DOI:** 10.3390/microorganisms8101465

**Published:** 2020-09-24

**Authors:** Daleniece Higgins, Nabanita Mukherjee, Chandan Pal, Irshad M. Sulaiman, Yu Jiang, Samir Hanna, John R. Dunn, Wilfried Karmaus, Pratik Banerjee

**Affiliations:** 1 Division of Epidemiology, Biostatistics, and Environmental Health, School of Public Health, University of Memphis, Memphis, TN 38152, USA; dhggins2@memphis.edu (D.H.); yjiang4@memphis.edu (Y.J.); karmaus1@memphis.edu (W.K.); 2 Department of Infectious Diseases, Center of Excellence for Influenza Research and Surveillance (CEIRS), St. Jude Children’s Research Hospital, Memphis, TN 38105, USA; nabanita.mukherjee@stjude.org; 3 Plant Health and Environment Laboratory, Ministry for Primary Industries, PO Box 2095, Auckland 1104, New Zealand; chandan.pal@mpi.govt.nz; 4 Southeast Food and Feed Laboratory, US Food and Drug Administration, Atlanta, GA 30309, USA; irshad.sulaiman@fda.hhs.gov; 5 Communicable and Environmental Diseases and Emergency Preparedness, Tennessee Department of Health, Nashville, TN 37243, USA; Samir.Hanna@tn.gov (S.H.); john.dunn@tn.gov (J.R.D.); 6 Department of Food Science and Human Nutrition, University of Illinois at Urbana-Champaign, Urbana, IL 61801, USA

**Keywords:** antibiotic resistance, virulence, *Salmonella*, virulotyping

## Abstract

The acquisition of antibiotic resistance (AR) by foodborne pathogens, such as *Salmonella enterica*, has emerged as a serious public health concern. The relationship between the two key survival mechanisms (i.e., antibiotic resistance and virulence) of bacterial pathogens is complex. However, it is unclear if the presence of certain virulence determinants (i.e., virulence genes) and AR have any association in *Salmonella*. In this study, we report the prevalence of selected virulence genes and their association with AR in a set of phenotypically tested antibiotic-resistant (*n* = 117) and antibiotic-susceptible (*n* = 94) clinical isolates of *Salmonella* collected from Tennessee, USA. Profiling of virulence genes (i.e., virulotyping) in *Salmonella* isolates (*n* = 211) was conducted by targeting 13 known virulence genes and a gene for class 1 integron. The association of the presence/absence of virulence genes in an isolate with their AR phenotypes was determined by the machine learning algorithm Random Forest. The analysis revealed that *Salmonella* virulotypes with gene clusters consisting of *avrA*, *gipA*, *sodC1*, and *sopE1* were strongly associated with any resistant phenotypes. To conclude, the results of this exploratory study shed light on the association of specific virulence genes with drug-resistant phenotypes of *Salmonella*. The presence of certain virulence genes clusters in resistant isolates may become useful for the risk assessment and management of salmonellosis caused by drug-resistant *Salmonella* in humans.

## 1. Introduction

*Salmonella* causes about 1.2 million illnesses, 23,000 hospitalizations, and 450 deaths in the United States every year [1,2]. Almost 1 million of those illnesses occur from foods contaminated with nontyphoidal *Salmonella* [1,3]. According to a 2019 estimate by the Centers for Disease Control and Prevention (CDC), drug-resistant nontyphoidal *Salmonella* caused more than 200,000 cases of illnesses and 70 deaths annually in the US [4]. Due to the emergence of antibiotic resistance (AR), treating nontyphoidal salmonellosis has become increasingly more difficult and sometimes impossible. In the United States alone, two million people are infected with antibiotic-resistant bacteria annually, and 23,000 people die consequently [5]. Furthermore, it is predicted that AR will cause a loss of up to $100 trillion to the global economy due to premature deaths [6].

Bacteria can acquire antibiotic resistance through multiple mechanisms, horizontal gene transfer being the main mechanism [1,7]. According to The National Antimicrobial Resistance Monitoring System (NARMS) 2015 report [8], important nontyphoidal *Salmonella* multidrug-resistant (MDR) phenotypes include resistance to ampicillin, chloramphenicol, streptomycin, sulfonamide (sulfamethoxazole/sulfisoxazole), and tetracycline (ACSSuT) and ACSSuT resistance, plus at least amoxicillin-clavulanic acid and ceftriaxone (ACSSuTAuCx). The report from NARMS [8] also finds that resistance to ciprofloxacin has increased in *Salmonella* since 1996. MDR *Salmonella* serotype l 4, [5], 12:i:- in human isolates increased from 18% in 2011 to 46% in 2013. There is also a growing rate of resistance of nontyphoidal *Salmonella* to nalidixic acid [3]. In addition, not only is *Salmonella* highly resistant to antibiotics, but unlike other bacteria, such as *Listeria* or *E. coli*, the majority of *Salmonella* are pathogenic to humans and/or animals [9]. It is yet to be explained if the increase of AR in *Salmonella* has increased the virulence potential of this bacteria or vice versa.

To date, there is no scientific consensus on the relationship between AR and virulence [1,10]. Consequently, it has not yet been explained if the increase of AR does also increase virulence determinants (expressions of specific virulence genes) of highly pathogenic bacteria, such as *Salmonella* [1,11,12]. What is known is that for pathogenic bacteria, both AR and virulence are necessary for survival under adverse conditions [1]. Specifically, the acquisition of AR is crucial for bacteria to adapt and survive in adverse environments containing antibiotics, and virulence genes are essential to overcome the host defense systems [1,13]. Furthermore, phenotypic changes that confer AR may be associated with decreased virulence, but also, there are reports of the opposite: that resistance can enhance virulence [13]. Bacteria may be able to increase effectiveness and repair injury by itself by using virulence determinants when in an environment with antibiotics to avoid host defense systems in host–pathogen interaction, suggesting a possibility of increased virulence [1,14]. Contrarily, other studies report that resistance mechanisms have a fitness cost that can weaken the bacteria when it comes to interacting with hosts by decreasing virulence [13,15]. However, in acquiring AR through horizontal gene transfer via mobile genetic elements, such as integrons, there are indications that AR genes can be silenced at no biological cost until needed, while other adaptive traits continue to be expressed, which would have no effect on virulence [15]. Thus, these considerations suggest that the development of AR is essential to enable pathogenic bacteria, such as *Salmonella*, to overcome antimicrobial therapies with an unknown consequence to their virulence.

Some previous studies examining the association between antibiotic resistance and virulence have found that when *Salmonella* acquires antibiotic resistance, the pathogen decreases in virulence potential [16,17,18,19,20]. In contrast, other studies have found that antibiotic resistance in *Salmonella* increases the virulence potential of the pathogen [21,22]. At the same time, another set of studies has found that the acquisition of resistance has no cost to *Salmonella* due to compensatory mutations [23,24,25,26]. In this study, our aim was to examine the association of specific virulence gene(s) with drug-resistant phenotypes of *Salmonella* isolates. To achieve this, we performed a molecular analysis-based virulotyping of selected virulence genes in a set of *Salmonella* clinical isolates displaying a range of phenotypic AR. Then, the molecular and AR phenotyping data were analyzed by a series of statistical and computational methods to find the association.

## 2. Materials and Methods

### 2.1. Samples (Salmonella Clinical Isolates)

Cultured confirmed *Salmonella* clinical isolates were obtained from the Tennessee Department of Health State Public Health Laboratory (TDH-SPHL). Isolates were collected from patients in Tennessee having a diagnosis of salmonellosis. Over two hundred isolates were randomly selected by TDH and sent to the University of Memphis School of Public Health Laboratory for analysis. All isolates were stored in −80 °C until use. The isolates were routinely cultured in Brain Heart Infusion (BHI) broth and plated on BHI agar at 37 °C with an incubation time of 18–24 h.

### 2.2. Genomic DNA Extraction

A microbial DNA isolation Kit, UltraClean^®^ Microbial DNA Isolation Kit (MO BIO Laboratories Inc., Carlsbad, CA, USA), was used to extract genomic DNA from each isolate following the manufacturer’s protocol. A NanoDrop^TM^ spectrophotometer (Thermo Fisher Scientific, Wilmington, DE, USA) was used to assess the DNA purity and quantify the concentration of DNA for each isolate. DNA was stored at −20 °C for later use of comparison between resistance and virulence genes.

### 2.3. Determination of Antibiotic Resistance

The antimicrobial susceptibility test was conducted to test the patterns of susceptibility in *Salmonella* isolates from the NARMS dataset by using the Clinical and Laboratory Standard Institute (CLSI) broth microdilution method of the Sensititre^TM^ system [27]. A list of antibiotics (*n* = 12) were tested in this process for all isolates, namely amoxicillin/clavulanic acid, ampicillin, azithromycin, cefoxitin, ceftiofur, ceftriaxone, chloramphenicol, ciprofloxacin, gentamicin, nalidixic acid, streptomycin, and tetracycline. For in-depth analysis, these antibiotics were also grouped into six different classes, namely beta-lactams (amoxicillin/clavulanic acid, cefoxitin, ceftiofur, ceftriaxone, and ampicillin), macrolides (azithromycin), chloramphenicols (chloramphenicol), fluoroquinolones (ciprofloxacin and nalidixic acid), aminoglycosides (gentamicin and streptomycin), and tetracyclines (tetracycline).

### 2.4. Virulotyping by Polymerase Chain Reaction (PCR)

Virulence gene profiling (i.e., virulotyping) in *Salmonella* isolates (*n* = 211) was done by end-point Polymerase Chain Reaction (PCR) targeting 13 selected virulence genes (*avrA*, *bcfC*, *gipA*, *invA*, *mgtC*, *pefA*, *sefA*, *siiD*, *sodC1*, *sopB*, *sopE1*, *spvC*, and *ssaQ*). We also tested an integron-associated integrase class 1 (*intI1*) gene marker in these isolates. Five virulence gene targets (*avrA*, *ssaQ*, *mgtC*, *siiD*, and *sopB*) were located on the *Salmonella* pathogenicity islands (SPIs) 1–5, three targets (*gipA*, *sodC1*, and *sopE1*) were located on prophages, one (*spvC*) was located on the *S.* Typhimurium virulence plasmid, and one (*bcfC*) was located on a fimbrial cluster [28]. Another target virulence gene, *invA*, is an invasion gene of the genus *Salmonella*; *sefA* is a fimbrial antigen of *S.* Enteritidis, and *pefA* is a plasmid-encoded fimbria of *S.* Typhimurium [29]. The integron-associated class 1 integrase gene (*IntI1)* is often located on transposons containing two to eight gene cassettes encoding resistance to a broad spectrum of antibiotics [15].

Multiplex PCR was used to amplify the DNA of each *Salmonella* isolate targeting the 13 virulence genes and integron-associated class 1 integrase gene (*IntI1*). Primers and DNA sequences of the genes can be found in Supplementary Table S1. Nine PCRs were completed based on the base-pair size of the primer and annealing temperature associated with the virulence gene. The virulence genes *avrA*, *mgtC*, and *sopB* were included in the first multiplex PCR. In the PCR reaction, 5 μL of genomic DNA template was added to 25 μL of 2× PCR Master Mix (Promega, Madison, WI, USA), 2 μL of nuclease-free water, and 1 μL of each primer (10 μM) [28]. The cycling conditions were as follows: 95 °C for 1 min, followed by 30 cycles of 95 °C for 30 s, 58 °C for 30 s, and 72 °C for 30 s, ending with a final extension step at 72 °C for 4 min and followed by a hold at 4 °C. Separate multiplex PCRs were completed for combinations of (*gipA* and *sopE1*), (*sodC1*, *spvC*, and *bcfC*), and (*ssaQ* and *siiD*) using the same cycling conditions. Another multiplex PCR was completed for *invA* and *sefA*. The reaction had a final volume of 25 μL containing PCR reaction buffer (50 mM KCl, 10 mM Tris-HCl, 2.5 mM MgCl_2_, pH 8.3), 200 μM dNTPs, 0.2 μM *invA* primers, 0.2 μM *sefA* primers, and 0.5 μM *pefA* primers, 2.5 U of Taq DNA polymerase (MBI Fermentas), and 0.2 μL of DNA template [29]. The cycling conditions consisted of denaturation for 30 s at 94 °C, annealing for 1 min at 55 °C, and extension for 1 min at 72 °C for 35 cycles, followed by a final extension for 7 min at 72 °C. The target virulence gene *pefA* followed the same conditions in a separate PCR. The final PCR targeted the integron-associated integrase class 1 with the following cycling conditions: denaturation at 94 °C for 3 min; 94 °C for 30 s, 60 °C for 30 s, 72 °C for 60 s, 35 cycles; 72 °C for 5 min [30]. There were two negative controls for each PCR with a template consisting of nuclease-free water. Gel electrophoresis of amplicons was completed using a 2% agarose gel containing 0.5 μg/mL of ethidium bromide to check the integrity of the DNA. A UV transilluminator was used to visualize the amplified DNA fragments for analysis.

### 2.5. Statistical and Computational Analysis

Our aim in this research was to examine how AR and virulence genes are associated with *Salmonella.* We examined the antibiotic susceptibility against 12 antibiotics from 6 antibiotic classes, and we associated that with the presence/absence of a set of 13 virulence genes in each isolate of *Salmonella*. The association of each virulence gene with AR phenotypes was determined by Pearson’s chi-square or Fisher’s exact test using SAS. Pearson’s chi-square and Fisher exact tests were performed to determine which antibiotic resistance was associated with specific virulence genes. Fisher’s exact test was used when the expected cell counts held less than five isolates. This analysis also provided information to determine which virulence genes were highly associated with MDR *Salmonella* isolates. To find out the set of important virulence genes that predict AR phenotypes, we used a machine learning algorithm Random Forest via ‘randomForest’ R package.

Subsequently, a network was built connecting phenotypical drug resistance and virulence genes to investigate co-occurrence patterns and identify combinations that are common among the *Salmonella* isolates. The network was created to visualize coinciding connections that can give information on patterns of frequency and incidence of virulence genes and drug resistance. The networks were illustrated using Cytoscape (version 3.5.1), which is an open-source software project for exploring, visualizing, and integrating biomolecular interaction networks into a conceptual framework [31,32].

Random Forest analysis was used to predict associations between virulence genes and AR phenotypes. The analysis uses classification trees to determine which variable is more important in determining the outcome by producing an importance score [33]. Thus, this analysis took all virulence genes into account and ranked different virulence genes in terms of their association with antibiotic resistance status in *Salmonella*. The Random Forest completes this analysis by quantifying the importance of each virulence gene in relation to resistance status, creating a score for variable importance that ranks each variable by disrupting the dependence between the variable and the response and measuring the change in the tree votes compared to the original observations, which can be standardized by dividing by the standard error derived from the between-tree variance [33,34]. Furthermore, variable importance scores can take into account interactions among variables without requiring model specification [34]. Particularly in our analysis, Random Forest determines which gene is more important to increase the probability of phenotypical resistance in *Salmonella* isolates. All statistical analysis was conducted in SAS version 9.4 (SAS Institute Inc., Cary, NC, USA) and R version 3.5.3 [35]. Statistical significance was set at *p* < 0.05.

## 3. Results

### 3.1. Profiling of Phenotypical Antibiotic Resistance and Virulotypes

A total of 211 *Salmonella* isolates were analyzed to determine the phenotypical antibiotic resistance and virulotype. Nearly half of the *Salmonella* isolates were pan-susceptible (*n* = 94, 45%) to the tested antibiotics. Only 117 (55%) isolates showed phenotypical antibiotic resistance. Of the isolates that showed resistance, 30 (26%) were single drug-resistant, and 87 (74%) were multidrug-resistant. Out of the multidrug-resistant isolates, 52 (60%) were resistant to 2–5 drugs, while 35 (40%) were resistant to more than five drugs. Interestingly, *Salmonella* isolates were more commonly resistant to azithromycin (*n* = 90, 43%), tetracycline (*n* = 73, 35%), and streptomycin (*n* = 67, 32%) than other tested drugs, as displayed in Supplementary Figure S1A. When individual drugs were grouped into antibiotic classes, *Salmonella* isolates were observed to be mostly resistant to macrolides (*n* = 90, 43%), tetracyclines (*n* = 73, 35%), and aminoglycosides (*n* = 69, 33%), as shown in Supplementary Figure S1B.

**Figure 1:**
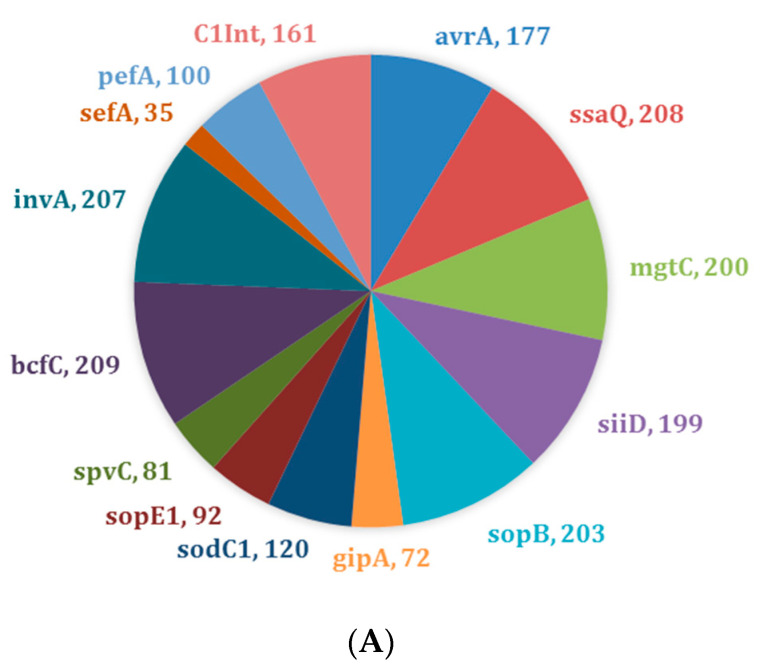

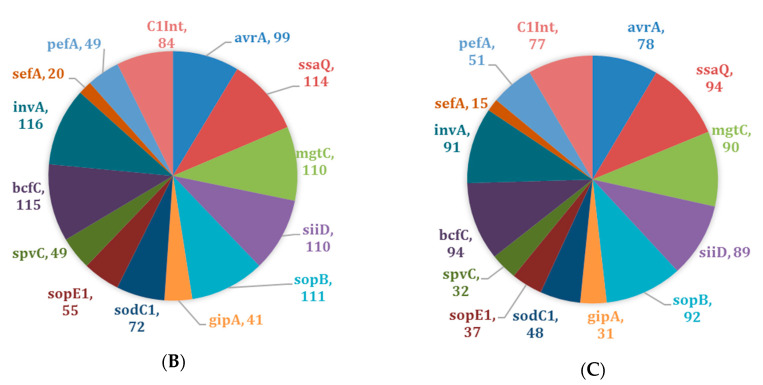
Distribution of virulence genes across *Salmonella* isolates (*n* = 211). (**A**) Prevalence of virulence genes across all *Salmonella* isolates; (**B**) Prevalence of virulence genes across drug-resistant *Salmonella* isolates only (*n* = 117); (**C**) Prevalence of virulence genes across drug-susceptible *Salmonella* isolates only (*n* = 94).

Virulence profiling (i.e., virulotyping) by PCR was used to identify targeted virulence genes in *Salmonella* isolates. The virulence genes *bcfC* (*n* = 209, 99%), *ssaQ* (*n* = 208, 99%), and *invA* (*n* = 207, 98%) were present in almost all isolates irrespective of their AR profiles (Figure 1A). A similar pattern was observed for drug-resistant isolates (*n* = 117) with genes *bcfC* (*n* = 115, 98%), *ssaQ* (*n* = 114, 97%), and *invA* (*n* = 116, 99%) (Figure 1B). There was a slight difference in the most common virulence genes observed in drug-susceptible isolates (*n* = 94), *bcfC* (*n* = 94, 100%), *ssaQ* (*n* = 94, 100%), and *sopB* (*n* = 92, 98%) (Figure 1C).

### 3.2. Statistical Association of Phenotypical Antibiotic Resistance Status with Virulence Genes

The association between targeted virulence genes and any antibiotic resistance was estimated by Pearson’s chi-square and Fischer’s exact tests (Table 1). Pearson’s chi square test did not show significant association between any antibiotic resistance status and virulence genes across all isolates, but significant differences were found between AR and a set of virulence genes or integron-associated integrase class I gene when only MDR isolates were considered (Table 2). Specifically, virulence genes *sefA* [χ^2^ (df = 3) = 11.15, *p* = <.0001], *siiD* [χ^2^ (df = 3) = 2.77, *p* = 0.008], *sodC1* [χ^2^ (df = 3) = 13.18, *p* = 0.004], *sopB* [χ^2^ (df = 3) = 4.42, *p* = 0.008], *ssaQ* [χ^2^ (df = 3) = 4.48, *p* = 0.030], *spvC* [χ^2^ (df = 3) = 8.63, *p* = 0.035], *mgtC* [χ^2^ (df = 3) = 3.27, *p* = 0.008], and *avrA* [χ^2^ (df = 3) = 0.981, *p* = 0.005], and a Class 1 integron gene [χ^2^ (df = 3) = 12.85, *p* = <0.0001] were significantly associated with MDR status (Table 2). In further analysis, no significant association was observed between AR and virulence genes in single drug-resistant isolates or 2–5 drug-resistant isolates (data not shown). However, there was a significant relationship between antibiotic resistance and various virulence genes in *Salmonella* isolates, which were resistant to more than 5 of the drugs. Specifically, isolates resistant to more than 5 drugs were significantly associated with *sefA* [χ^2^ (df = 1) = 5.18, *p* = 0.023], *sodC1* [χ^2^ (df = 1) = 10.71, *p* = 0.001], *sopE1* [χ^2^ (df = 1) = 4.39, *p* = 0.036], *spvC* [χ^2^ (df = 1) = 7.10, *p* = 0.008], *pefA* [χ^2^ (df = 1) = 6.75, *p* = 0.010], and one integron-associated integrase class 1 gene [χ^2^ (df = 1) = 10.23, *p* = 0.001] (Table 3).

### 3.3. Network Analysis of Phenotypical Drug Resistance and Virulence Genes

**Figure 2:**
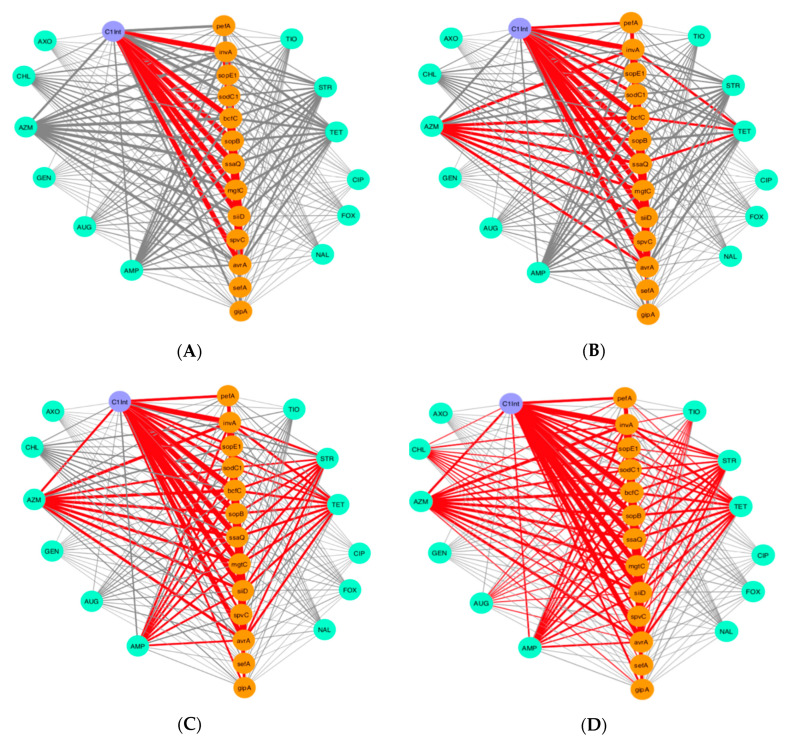
Visualization of co-occurrence pattern of phenotypical drug resistance and prevalence of virulence genes across *Salmonella* isolates (*n* = 211). No connections are shown between drug (i.e., antibiotic) resistance genes to better emphasize connections between drug resistance and virulence. The red lines (e.g., edge) indicate a high incidence of connections that occur together between drug resistance and virulence among the isolates. The thickness of the line between nodes denotes the frequency of isolates that share the same coinciding connections. Nodes in cyan, orange, and purple are antibiotic resistance, virulence genes, and the integron-associated integrase gene, respectively. (**A**) Observed connections between drug resistance and virulence genes across 100 out of 211 isolates. (**B**) Observed connections between drug resistance and virulence genes across 70 isolates out of 211 isolates. (**C**) Observed connections between drug resistance and virulence genes across 50 isolates out of 211 isolates. (**D**) Observed connections between drug resistance and virulence genes across 25 isolates out of 211 isolates. Key: amoxicillin/clavulanic acid (AUG), ampicillin (AMP), azithromycin (AZM), cefoxitin (FOX), ceftiofur (TIO), ceftriaxone (AXO), chloramphenicol (CHL), ciprofloxacin (CIP), gentamicin (GEN), nalidixic acid (NAL), streptomycin (STR), tetracycline (TET).

To investigate the association of phenotypical drug resistance and virulence genes across *Salmonella* isolates, we generated a co-occurrence network of connections between phenotypic drug resistance and virulence genes that were observed among the 211 isolates. In Figure 2, we displayed four distinct networks that display the connections between phenotypic drug resistance along with the presence/absence of certain virulence genes in each isolate, resulting in specific combinations that are commonly observed among the *Salmonella* isolates. We observed the co-existence of certain drug resistance with certain virulence genes in the same isolate more frequently than others. In nearly half of the isolates (*n* = 100), there was a high frequency of integron-associated integrase class I gene and the following virulence genes: *invA*, *bcfC*, *sopB*, *ssaQ*, *mgtC*, *siiD*, and *spvC* (Figure 2A). Furthermore, the association between certain drug resistance (azithromycin and tetracycline) and the presence of virulence genes was also frequent in 1/3 of the tested isolates (Figure 2B). Similarly, when we looked into the combination of AR and virulence genes that were present in nearly 1/4 of the 50 isolates, we observed a further association between resistance to the aforementioned drugs (azithromycin and tetracycline), streptomycin, and ampicillin with multiple virulence genes (Figure 2C). Lastly, across 25 isolates (12% of the isolates), phenotypic resistance to three more drugs, chloramphenicol, ceftiofur, and amoxicillin/clavulanic acid, was observed along with the presence of virulence genes (Figure 2D). Thus, it is possible that having the mobile genetic element integron, or resistance to azithromycin, tetracycline, streptomycin, and ampicillin drugs can lead to an increase in the presence of virulence.

### 3.4. Predictive Analysis of Drug Resistance as Indicated by Virulence Genes by Random Forest

**Figure 3:**
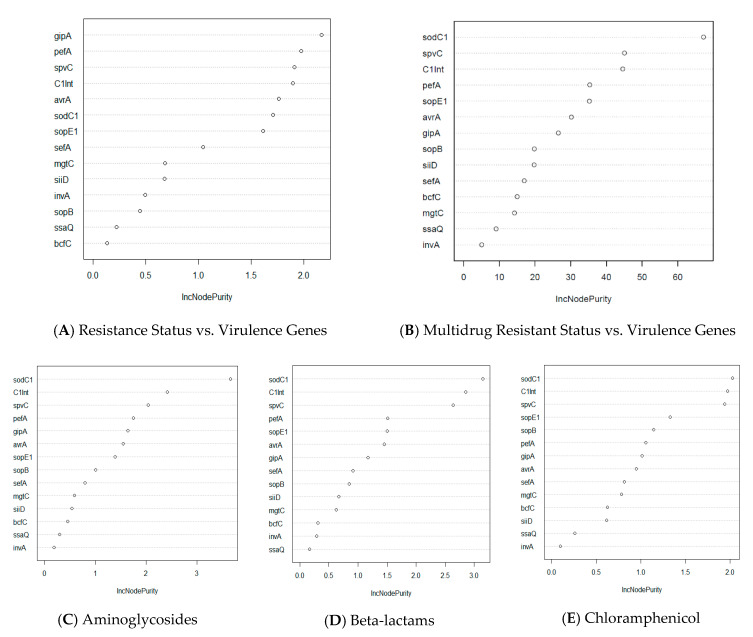

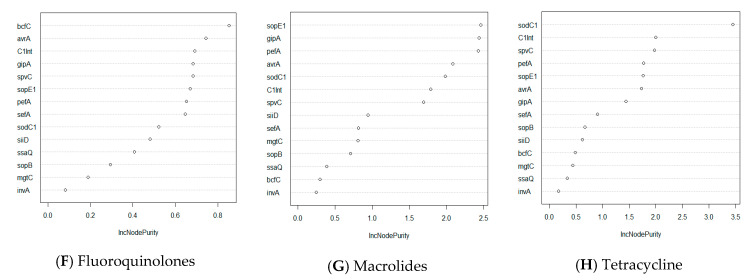
Random Forest analysis of association of virulence genes with resistance status by antibiotic class in *Salmonella* isolates (*n* = 211). The y-axis indicates the importance score of each virulence gene. Virulence genes that are at top of the graph are the most important genes in determining resistance status (multidrug resistance, resistance to specific class of antibiotics, etc.). (**A**) Prediction of important virulence genes for all isolates that show any drug resistance. (**B**) Prediction of important virulence genes for isolates that are resistant to more than one drug. (**C**–**H**) Prediction of important virulence genes for all isolates that have resistance to a specific class of drug.

We explored further this relationship by utilizing a machine learning approach, using the Random Forest algorithm, to predict which virulence genes could better indicate (i.e., used as a proxy) for the potential presence of certain types of drug resistance. The Random Forest analysis shows the top three virulence genes that are associated with antibiotic resistance include *gipA*, *pefA*, and *spvC* (Figure 3A). Among these genes, *gipA*, a bacteriophage-associated virulence gene, has the highest important score in the prediction of overall antibiotic resistance status and the prevalence of virulence genes.

Furthermore, one non-SPI gene, *sodC1*, was found to be the most important driver for multidrug resistance and in antibiotic classes, namely beta-lactams, chloramphenicol, aminoglycosides, and tetracycline (Figure 3B–E,H). Antibiotic classes of fluoroquinolones and macrolides differ from the aforementioned classes in that the most important virulence genes that drive drug resistance for these classes are *bcfC*, which is located in the chromosome, and *sopE1*, another bacteriophage-associated gene, respectively (Figure 3F,G). When investigating the drug resistance of individual *Salmonella* isolates, we found that one integron-associated integrase class I gene (*IntI1*) was found to be significant for the prediction of phenotypical resistance of the isolates to amoxicillin/clavulanic acid, ceftiofur, and cefoxitin (Supplementary Figure S2). Similarly, one virulence gene, *sodC1*, was found to be the most important predictor for resistance to four drugs, namely ampicillin, chloramphenicol, streptomycin, and tetracycline. Interestingly, although the *spvC* gene did not have the highest importance score in predicting for any of the drugs, this gene was still found to be one of the three most important variables for phenotypical drug resistance to seven drugs tested, namely ampicillin, amoxicillin/clavulanic acid, chloramphenicol, cefoxitin, gentamycin, nalidixic acid, and tetracycline (Supplementary Figure S2).

## 4. Discussion

We analyzed 211 clinical isolates of *Salmonella* from the cases of reported salmonellosis patients across the state of Tennessee, USA. This study shows that the status of selected virulence genes did not significantly differ between drug-resistant and drug-susceptible *Salmonella* isolates that were tested, but it does differ between multidrug-resistant and drug-susceptible isolates. Specifically, phenotypical antibiotic resistance may not increase the virulence potential of *Salmonella* (as assessed by the virulence gene profiling) with isolates that are single drug-resistant. However, it is possible that virulence may increase in isolates that are multidrug-resistant. This may result in the reduction of the overall efficacy of conventional antibiotics that are generally used to counteract infections caused by MDR *Salmonella* with higher carriage of virulence genes. Our study reveals that resistance to conventional antibiotics, such as azithromycin, tetracycline, and streptomycin (Supplementary Figure S1), is common, which reduces the chances of cure for infected patients by these antibiotics. Moreover, these antibiotics exist in the market for a prolonged period with more widespread usage; therefore, greater resistance can be expected.

The intricacy of the relationship between antibiotic resistance and virulence, the two critical survival mechanisms in bacteria, is not yet fully understood. There is substantial evidence in the scientific literature that bacterial fitness costs, such as diminished growth rates and virulence, may be attributed to the acquisition of resistance by the overexpression of genes responsible for antibiotic resistance or multidrug resistance efflux pumps [36,37]. Studies have also shown a reduced survival of drug-resistant strains in the absence of antibiotic selective pressure, indicating a severe impairment of fitness [19]. On the contrary, several other studies reported that the acquisition of drug resistance enhanced the fitness and virulence of pathogens [13,38]. Compensatory mutations restoring fitness is suggested as a possible mechanism that stabilizes resistance to the original level [26,39]. In our study, we did not find any significant “fitness cost” that is associated with the loss of virulence potentials in multidrug-resistant phenotypes or vice versa.

Our findings are in agreement with past studies that reported high levels of resistance to conventional antibiotics such as tetracycline and streptomycin, but we found more susceptibility to third-generation cephalosporins, including ceftriaxone, to counteract the infection [40,41]. Furthermore, by analyzing antibiotics to which *Salmonella* isolates are more resistant or susceptible, we were able to determine a possible connection between the virulence determinants and antibiotic resistance.

We found a high prevalence of virulence genes *bcfC*, *ssaQ*, and *invA* in both drug-susceptible and drug-resistant isolates (Figure 1). As the majority of *Salmonella* virulence genes are located on *Salmonella* pathogenicity islands (SPIs) [42,43], it is expected that *ssaQ*, a gene located on an SPI that functions as a secretion system apparatus protein, is present in most of the isolates we analyzed. One invasion-related virulence gene, *invA*, was also expected to be present in the majority of the isolates, as previous studies have also found this gene to be present in most isolates [44,45,46]. Furthermore, the *invA* gene is found to be conserved among the *Salmonella* serotype [44]. In this context, it is important to note that the apparent absence of the *invA* gene in some isolates may be due to the allelic dropout as a result of a single-nucleotide polymorphism (SNP) in the annealing region of the primers or due to the lack and/or mutation in the gene itself [47,48]. In addition to virulence genes *invA* and *ssaQ*, another virulence gene *bcfC*, which is located in the chromosome, was also expected, and these results agree with previous studies [49,50,51].

Interestingly, of the three most prominent virulence genes, only *ssaQ* was found to be significantly associated with MDR *Salmonella* isolates, using both Pearson’s χ^2^ and Exact χ^2^ tests, along with other virulence genes (Table 2). Since *ssaQ*, *mgtC*, *siiD*, *sopB*, and *avrA* are located on the SPIs, it was expected that these genes might be significantly associated with resistance. Virulence genes such as *avrA*, which facilitates bacterial proliferation and intracellular survival [52,53], or *mgtC*, which facilitates bacterial growth under adverse conditions [54], can lead to a decreased susceptibility to certain drugs. Furthermore, virulence genes such as *sopB*, a translocated effector protein, can use genes such as *sopE1*, which also functions as an effector protein, to promote bacterial entry and facilitation of the invasion process [55]. Furthermore, we also expected a significant relationship between the class 1 integron gene and antibiotic resistance, as integron gene cassettes are known to carry genes that encode resistance to antibiotics.

The co-occurrence network (Figure 2) displayed drugs to which *Salmonella* isolates are commonly resistant and their linkage to virulence genes. Thus, there is a higher potential for the isolate to have a higher prevalence of virulence genes if it shows phenotypical resistance to antibiotics such as azithromycin, tetracycline, streptomycin, or ampicillin. When we examined the relationship between drug resistance and virulence genes by different methods, we observed that our network visualization is in agreement with the Random Forest analysis. In the Random Forest analysis, we predicted the importance of each virulence gene to determine which gene is more important to increase the probability of phenotypical resistance in *Salmonella* isolates. We found that bacteriophage-associated virulence genes *sodC1* and *gipA* are the most important predictors of determining phenotypical resistance in *Salmonella*. Specifically, one non-SPI gene, *sodC1*, was found to be an important driver for resistance to antibiotic classes, beta-lactams, chloramphenicol, aminoglycosides, and tetracycline, and a set of individual antibiotics, such as ampicillin, streptomycin, and tetracycline (Figure 3; Supplementary Figure S2). The Pearson’s χ^2^ and Exact χ^2^ tests also found a significant relationship between *sodC1* and multidrug resistance in *Salmonella* isolates (Table 2). This result was expected, since the presence of *sodC1* was found more in drug-resistant isolates by 10%. Moreover, *sodC1* encodes a periplasmic Cu–Zn superoxide dismutase that promotes bacterial survival in macrophages and has been found to be associated with resistance to specific antibiotics [56,57,58].

Interestingly, *gipA*, a bacteriophage-associated virulence gene that encodes Peyer’s patch-specific virulence factor, is also an important gene that revealed a strong association of overall antibiotic resistance status and the prevalence of virulence genes. Although this virulence gene was not the most important driver for any specific AR that we studied, it is known as a critical component for survival bypassing the host immune system in *Salmonella* [59]. As this gene enables bacteria to withstand certain stress, it can also be predicted to be associated with an increase in resistance. In addition to the previously mentioned bacteriophage-associated virulence genes, one integron-associated marker gene (*IntI1*) is consistently found to be included as one of the three most important predictor genes in both single and multidrug-resistant isolates. This result agrees well with previous reports that integron-associated marker genes are highly associated with antibiotic resistance [1,15,31,60]. Thus, our findings of the co-occurrence of the integron-associated integrase class I gene with antibiotic resistance and as a major predictor (as revealed by the Random Forest data) of resistance for multiple antibiotics is consistent with previous reports of integrons being associated with antibiotic resistance [61,62,63].

Rather than detecting the presence/absence of antibiotic-resistant genes, we assessed the relationship between antibiotic resistance (AR) and virulence genes in *Salmonella* isolates by considering phenotypical drug resistance. The data of phenotypical resistance provide a better insight into “true AR” that is clinically relevant. The presence of antibiotic resistance genes that are not expressed or partially expressed often gives positive genotypic results. However, bacteria containing these genes (that are not expressed) may still remain phenotypically susceptible, making the result a false positive [64]. Therefore, evaluating phenotypical AR against virulence genes helped us better characterize the association, which was one of the strengths of this study.

In the current study, we did not perform serotyping of the culture-confirmed *Salmonella* isolates. With the advancement of gene sequencing methodologies, the need for resource-intensive antiserum-based serotyping is receding [65]. Traditional serotyping may be replaced by more robust in silico platforms such as SeqSero, which allows the integration of the *Salmonella* classical serotyping scheme into a whole-genome sequencing (WGS)-based serotyping inference [66]. Recent genomics-based studies reveal that the attribution of phenotypical characteristics to individual *Salmonella* strains or serovars is not enough to improve the hazard characterization [67]. Studies have shown that the genomics data (without serotyping) of *Salmonella* isolates can efficiently be used in epidemiological and prevalence-related risk assessments [65,68]. Moreover, a recent paper reports that the variation in the phenotypical characteristics (such as in vitro virulence) among individual strains from the same serovar is more significant than that found between serovars [67]. Therefore, the lack of serotype information in our study does not confound our findings of the relationship between phenotypic AR and virulence genes.

One potential limitation in our study is that due to the nature of the data, some error rates for the drug category prediction exceeded 10% in the Random Forest analysis. Furthermore, we analyzed a specific set of antibiotics (12 antibiotics from six classes) based on their availability. In addition, we have chosen a small sample of virulence genes (13) based on literature findings regarding *Salmonella* virulence. Thus, we could only determine the association between resistance and virulence prevalence for a small set of antibiotics and virulence genes. Including more antibiotics and other known virulence genes from *Salmonella* would provide a more in-depth association between AR and virulence genes in future studies. However, a major strength in the present study is the use of Random Forest analysis to predict the importance of each virulence gene in its relation to phenotypical antibiotic resistance. Thus, we were able to take the joint effects of virulence genes into account that may occur in the same isolate and the results in phenotypical resistance.

## 5. Conclusions

Our work presented here highlights the association between the distribution of bacterial virulence genes and their phenotypic drug-resistance pattern among *Salmonella* isolates from patients diagnosed with salmonellosis using statistical and computational methods. In addition, the distribution pattern of selected virulence genes did not significantly differ between resistant and susceptible *Salmonella* isolates, but it did differ between multidrug-resistant and susceptible isolates. Moreover, we have confirmed that virulence genes *sodC1* and *gipA*, as well as integrons, warrant a closer look with a wide selection of antibiotics to confirm an association that can lead to increased virulence in bacteria. The results of this study will be useful in exploring the relationship between the genetic character (such as the status of virulence genes) and the phenotypical traits (e.g., drug resistance and virulence). Therefore, our findings pave the path of future studies to find the causality and mechanism of pathogenesis and antibiotic resistance. Likewise, future studies involving antibiotic resistance genes profiling in tandem with sequence-based virulotyping and sequencing of the integron 1 gene can be undertaken to better examine the relationship between antibiotic resistance and virulence to devise useful strategies to better control the severity of salmonellosis in human.

## Figures and Tables

**Table 1:** Association between any antibiotic resistance and virulence genes in *Salmonella* isolates (*n* = 211).

Virulence Gene	Absence/Presence	Antibiotic Susceptible n (%)	Antibiotic Resistance n (%)	Chi-sq/Fischer Value	*p* Value
*arvA*	0	16 (47%)	18 (53%)	0.103	0.748
	1	78 (44%)	99 (56%)		
*bcfC*	0	0 (0%)	2 (100%)	1.622	0.306
	1	94 (45%)	115 (55%)		
*gipA*	0	63 (45%)	76 (55%)	0.099	0.753
	1	31 (43%)	41 (57%)		
*mgtC*	0	4 (36%)	7 (64%)	0.315	0.575
	1	90 (45%)	110 (55%)		
*sefA*	0	79 (45%)	97 (55%)	0.049	0.825
	1	15 (43%)	20 (57%)		
*siiD*	0	5 (42%)	7 (58%)	0.043	0.230
	1	89 (45%)	110 (55%)		
*sodC1*	0	46 (51%)	45 (49%)	2.33	0.127
	1	48 (40%)	72 (60%)		
*sopB*	0	2 (25%)	6 (75%)	1.29	0.161
	1	92 (45%)	111 (55%)		
*sopE1*	0	57 (48%)	62 (52%)	1.24	0.266
	1	37 (40%)	55(60%)		
*spvC*	0	62 (48%)	68 (52%)	1.35	0.245
	1	32 (40%)	49 (60%)		
*ssaQ*	0	0 (0%)	3 (100%)	2.45	0.169
	1	94 (45%)	114 (55%)		
*pefA*	0	43 (39%)	68 (61%)	3.20	0.074
	1	51 (51%)	49 (49%)		
*invA*	0	3 (75%)	1 (25%)	1.53	0.195
	1	91 (44%)	116 (56%)		
*Class 1 integron*	0	17 (34%)	33 (66%)	2.95	0.086
	1	77 (48%)	84 (52%)		

Absence = 0; Presence = 1.

**Table 2:** Association between multiple antibiotic resistance and virulence genes in *Salmonella* isolates (*n* = 211).

Virulence Gene	Absence/Presence	Antibiotic Susceptible n (%)	Single Drug Resistance n (%)	2–5 Drug Resistance n (%)	>5 Drug Resistance n (%)	Chi-sq/Fischer Value	*p* Value
*arvA*	0	16 (47%)	6 (18%)	8 (23%)	4 (12%)	0.981	0.005
	1	78 (44%)	24 (14%)	44 (25%)	31 (17)		
*bcfC*	0	0 (0%)	0 (0%)	1 (50%)	1 (50%)	3.07	0.082
	1	94 (45%)	30 (14%)	51 (24%)	34 (16%)		
*gipA*	0	63 (45%)	20 (15%)	32 (23%)	24 (17%)	0.612	0.893
	1	31 (43%)	10 (14%)	20 (28%)	11 (15%)		
*mgtC*	0	4 (36%)	0 (0%)	4 (36%)	3 (28%)	3.27	0.008
	1	90 (45%)	30 (15%)	48 (25%)	32 (16%)		
*sefA*	0	79 (45%)	26 (15%)	48 (27%)	23 (13%)	11.15	< 0.0001
	1	15 (43%)	4 (11%)	4 (11%)	12 (35%)		
*siiD*	0	5 (42%)	0 (0%)	4 (33%)	3 (25%)	2.77	0.008
	1	89 (45%)	30 (15%)	48 (24%)	32 (16%)		
*sodC1*	0	46 (51%)	17 (19%)	22 (24%)	6 (6%)	13.18	0.004
	1	48 (40%)	13 (11%)	30 (25%)	29 (24%)		
*sopB*	0	2 (25%)	0 (0%)	4 (50%)	2 (25%)	4.42	0.008
	1	92 (45%)	30 (15%)	48 (24%)	33 (16%)		
*sopE1*	0	57 (48%)	15(13%)	33 (27%)	14 (12%)	6.07	0.108
	1	37 (40%)	15 (16%)	19 (21%)	21 (23%)		
*spvC*	0	62 (48%)	21 (16%)	33 (25%)	14 (11%)	8.63	0.035
	1	32 (40%)	9 (11%)	19 (23%)	21 (26%)		
*ssaQ*	0	0 (0%)	0 (0%)	2 (67%)	1 (33%)	4.48	0.030
	1	94 (45%)	30 (15%)	50 (24%)	34 (16%)		
*pefA*	0	43 (39%)	17 (15%)	26 (23%)	25 (23%)	7.089	0.069
	1	51 (51%)	13 (13%)	26 (26%)	10 (10%)		
*invA*	0	3 (75%)	0 (0%)	1 (25%)	0 (0%)	2.10	0.087
	1	91 (44%)	30 (14%)	51 (25%)	35 (17%)		
*Class 1 integron*	0	17 (34%)	4 (8%)	13 (26%)	16 (32%)	12.85	< 0.0001
	1	77 (48%)	26 (16%)	39 (24%)	19 (12%)		

Absence = 0; Presence = 1.

**Table 3:** Association between >5 drug resistance and virulence genes in *Salmonella* isolates (*n* = 129).

Virulence Gene	Absence/Presence	Antibiotic Susceptible n (%)	>5 Drug Resistance n (%)	Chi-sq/Fischer Value	*p* Value
*arvA*	0	16 (47%)	4 (12%)	0.609	0.170
	1	78 (44%)	31 (17)		
*bcfC*	0	0 (0%)	1 (50%)	2.71	0.271
	1	94 (45%)	34 (16%)		
*gipA*	0	63 (45%)	24 (17%)	0.028	0.867
	1	31 (43%)	11 (15%)		
*mgtC*	0	4 (36%)	3 (28%)	0.926	0.200
	1	90 (45%)	32 (16%)		
*sefA*	0	79 (45%)	23 (13%)	5.18	0.023
	1	15 (43%)	12 (35%)		
*siiD*	0	5 (42%)	3 (25%)	0.464	0.236
	1	89 (45%)	32 (16%)		
*sodC1*	0	46 (51%)	6 (6%)	10.71	0.001
	1	48 (40%)	29 (24%)		
*sopB*	0	2 (25%)	2 (25%)	1.09	0.236
	1	92 (45%)	33 (16%)		
*sopE1*	0	57 (48%)	14 (12%)	4.39	0.036
	1	37 (40%)	21 (23%)		
*spvC*	0	62 (48%)	14 (11%)	7.10	0.008
	1	32 (40%)	21 (26%)		
*ssaQ*	0	0 (0%)	1 (33%)	2.71	0.271
	1	94 (45%)	34 (16%)		
*pefA*	0	43 (39%)	25 (23%)	6.75	0.010
	1	51 (51%)	10 (10%)		
*invA*	0	3 (75%)	0 (0%)	1.14	0.384
	1	91 (44%)	35 (17%)		
*Class 1 integron*	0	17 (34%)	16 (32%)	10.23	0.001
	1	77 (48%)	19 (12%)		

Absence = 0; Presence = 1.

## Supplementary Materials

The following are available online at https://www.mdpi.com/2076-2607/8/10/1465/s1, Figure S1: Antibiotic resistance distribution across all *Salmonella* isolates (*n* = 211), Figure S2: Random Forest analysis of association of virulence genes with resistance status by individual drug in *Salmonella* isolates (*n* = 210), Table S1: Virulence genes and the primers used for PCR-based virulotyping.

## Disclaimer

The findings and conclusions in this communication are those of the authors and do not necessarily represent the views or official position of the U.S. Food and Drug Administration (FDA) or the U.S. Department of Health and Human Services (DHHS) or the New Zealand Ministry for Primary Industries (MPI). The names of vendors or manufacturers are provided as examples of available product sources; inclusion does not imply endorsement of the vendors, manufacturers, or products by the FDA or the DHHS.
