# Supplementary material for: Association of Virulence and Antibiotic Resistance in Salmonella—Statistical and Computational Insights into a Selected Set of Clinical Isolates
Source: Microorganisms. 2020 Sep 24;8(10):1465. doi: 10.3390/microorganisms8101465 (PMC7598717; doi:10.3390/microorganisms8101465)
Supplement: Supplementary file 1 [file microorganisms-08-01465-s001.pdf]

Supplementary Figures

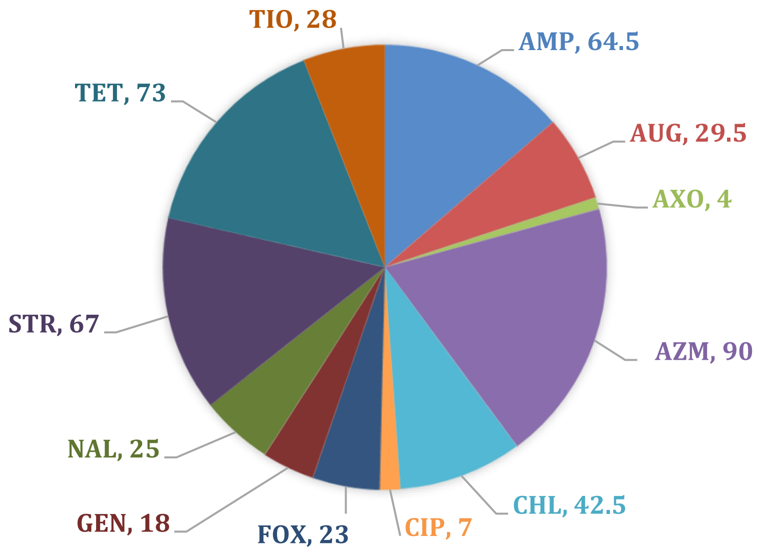

(A)

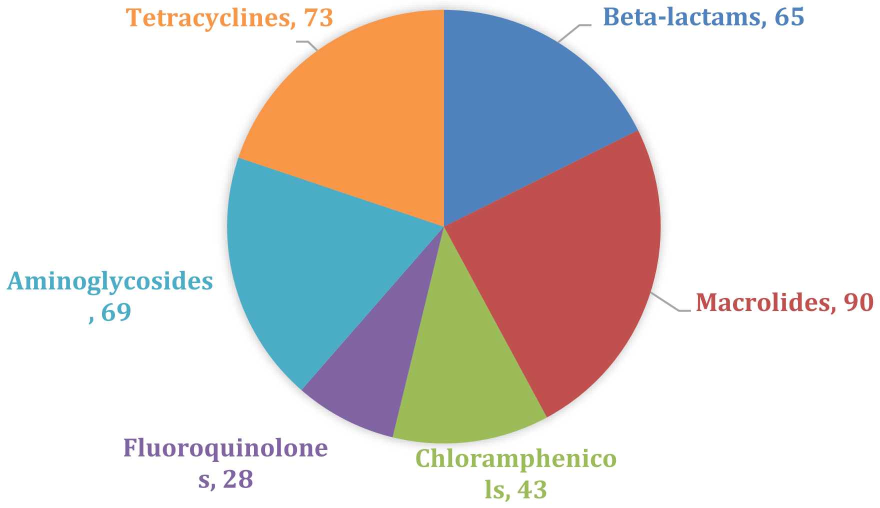

(B)

Figure S1. Antibiotic resistance distribution across all *Salmonella* isolates (n=211). A) Displays individual resistance status across all *Salmonella* isolates. B) Displays resistance status by class across all *Salmonella* isolates. **Key:** amoxicillin/clavulanic acid (AUG), ampicillin (AMP), azithromycin (AZM), cefoxitin (FOX), ceftiofur (TIO), ceftriaxone (AXO), chloramphenicol (CHL), ciprofloxacin (CIP), gentamicin (GEN), nalidixic acid (NAL), streptomycin (STR), and tetracycline (TET).

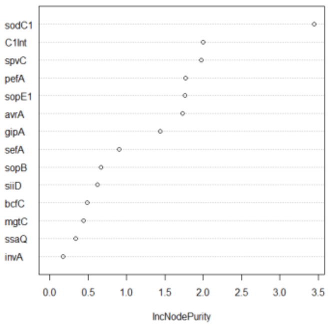

(A) Tetracyclines

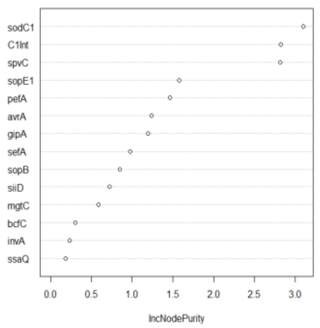

(B) Ampicillin

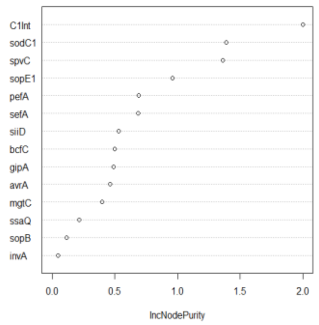

(C) Amoxicillin/Clavulanic Acid

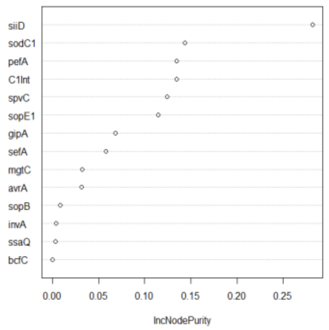

(D) Ceftriaxone

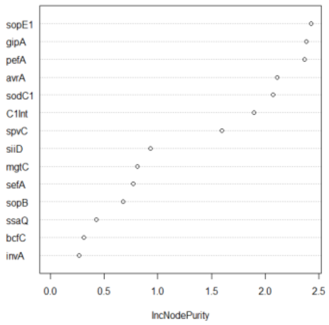

(E) Azithromycin

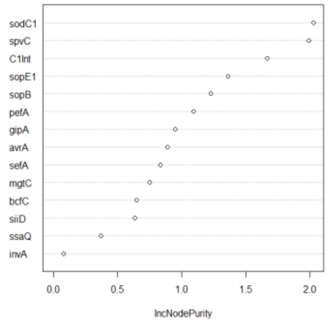

(F) Chloramphenicol

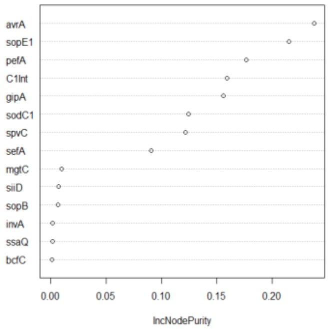

(G) Ciprofloxacin

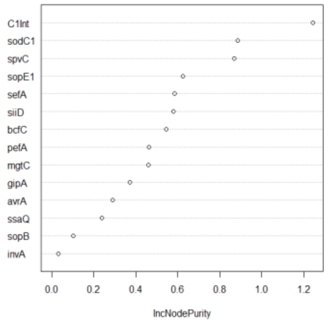

(H) Cefoxitin

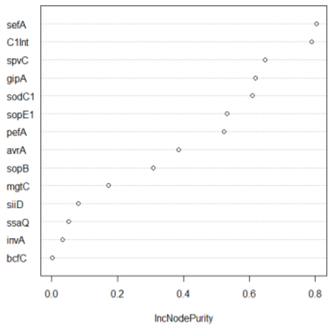

(I) Gentamicin

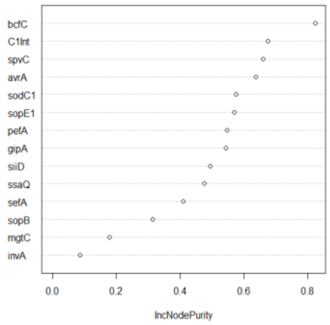

(J) Nalidixic Acid

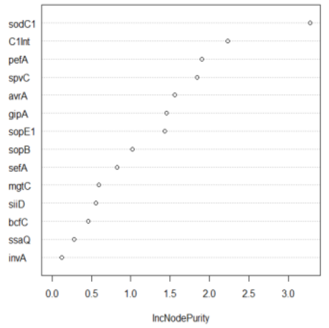

(K) Streptomycin

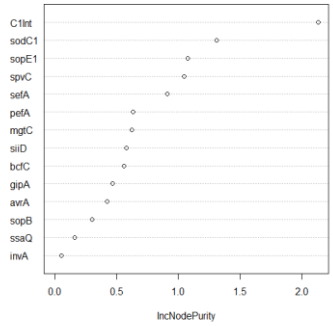

(L) Ceftiofur

Figure S2. Random Forest analysis of association of virulence genes with resistance status by individual drug in *Salmonella* isolates (n=210). Along the Y axis indicates the importance score of each virulence gene. Virulence genes which are at top of the graph are the most important genes in determining resistance status (multidrug resistance, resistance to specific class of antibiotics, etc.). **A-L)** Displays a prediction of important genes for all isolates that have phenotypical resistance to a specific drug.

Supplementary Table S1. Virulence genes and the primers used for PCR-based virulotyping.

Genes	Location	Function	Primer sequence (5′-3′)	Amplicon size (bp)	Reference
avrA	SPI-1, Centisome 63	Inhibits the key proinflammatory, antiapoptotic NF-kappa B pathway	CCTGTATTGTTGAGCGTCTGG<br>AGAAGAGCTTCGTTGAATGTCC	422	[1]
ssaQ	SPI-2, Centisome 31-30, 5	Secretion system apparatus protein, component of second T3SS	GAATAGCGAATGAAGAGCGTCC<br>CATCGTGTTATCCTCTGTCAGC	455	[1]
mgtC	SPI-3, Centisome 82	Intramacrophage survival protein	TGACTATCAATGCTCCAGTGAAT<br>ATTTACTGGCCGCTATGCTGTTG	677	[1]
siiD	SPI-4, Centisome 92	HLYD family secretion protein	GAATAGAAGACAAAGCGATCATC<br>GCTTTGTCCACGCCTTTCATC	655	[1]
sopB	SPI-5, Centisome 20	Translocated effector protein via T3SS	TCAGAAGRCGTCTAACCACTC<br>TACCGTCCTCATGCACACTC	517	[1]
gipA	Gifsy-1 bacteriophage	Peyer's patch-specific virulence factor	ACGACTGAGCAGGCTGAG<br>TTGGAAATGGTGACGGTAGAC	518	[1]
sodC1	Gifsy-2 bacteriophage	Periplasmic Cu, Zn-superoxide dismutases	CCAGTGGAGCAGGTTTATCG<br>GGTGCGCTCATCAGTTGTTC	424	[1]
sopE1	Cryptic bacteriophage	Translocated T3SS effector protein	CGGGCAGTGTTGACAAATAAAG<br>TGTTGGAATTGCTGTGGAGTC	422	[1]
spvC	Virulence pSLT plasmid	Spv region promotes rapid growth and survival within host	ACTCCTTGCACAACCAAATGCGGA<br>TGTCTTCTGCATTTCGCCACC	467	[1]
bcfC	Chromosome, Fimbrial gene cluster	Bovine colonization factor, fimbrial usher	ACCAGAGACATTGCCTTCC<br>TTCTGATCGCCGCTATTCG	467	[1]
invA	SPI-1	Triggers internalization required for invasion of deep tissue cells	TTGTTACGGCTATTTTGACCA<br>CTGACTGCTACCTTGCTGATG	521	[2,3]
sefA	Chromosome-encoded fimbrial operon	Encodes major subunit fimbrial protein of *S.* Enterica serotype	GCAGCGGTTACTATTGCAGC<br>TGTGACAGGGACATTTAGCG	330	[2,4]
pefA	Plasmid-encoded major fimbrial subunit	Fimbrial virulence gene of *S.* Typhimurium	TTCCATTATTGCACTGGGTG<br>GGCATCTTTCGCTGTGGCTT	497	[2,5]
**Class 1 Integrons**	*Salmonella* genomic island 1	Associated with a variety of resistance gene cassettes	CGAACGAGTGGCGGAGGGTG<br>TACCCGAGAGCTTGGCACCCA	312	[6,7]

Reference:

1. Huehn, S.; La Ragione, R.M.; Anjum, M.; Saunders, M.; Woodward, M.J.; Bunge, C.; Helmuth, R.; Hauser, E.; Guerra, B.; Beutlich, J., Virulotyping and antimicrobial resistance typing of *Salmonella enterica* serovars relevant to human health in Europe. *Foodborne Pathogens and Disease* **2010**, *7*, 523-535.
2. Cortez, A.; Carvalho, A.; Ikuno, A.; Bürger, K.; Vidal-Martins, A., Identification of *Salmonella* spp. isolates from chicken abattoirs by multiplex-PCR. *Research in veterinary science* **2006**, *81*, 340-344.
3. van der Velden, A.W.; Lindgren, S.W.; Worley, M.J.; Heffron, F., *Salmonella* pathogenicity island 1-independent induction of apoptosis in infected macrophages by Salmonella enterica serotype typhimurium. *Infect Immun* **2000**, *68*, 5702-5709.
4. Doran, J.L.; Collinson, S.K.; Clouthier, S.C.; Cebula, T.A.; Koch, W.H.; Burian, J.; Banser, P.A.; Todd, E.C.; Kay, W.W., Diagnostic potential of *sefA* DNA probes to *Salmonella* enteritidis and certain other O-serogroup D1 *Salmonella* serovars. *Mol Cell Probes* **1996**, *10*, 233-246.
5. Figueiredo, R.; Card, R.; Nunes, C.; AbuOun, M.; Bagnall, M.C.; Nunez, J.; Mendonca, N.; Anjum, M.F.; da Silva, G.J., Virulence Characterization of *Salmonella* enterica by a New Microarray: Detection and Evaluation of the Cytolethal Distending Toxin Gene Activity in the Unusual Host *S.* Typhimurium. *PLoS One* **2015**, *10*, e0135010.
6. Mulvey, M.R.; Boyd, D.A.; Olson, A.B.; Doublet, B.; Cloeckaert, A., The genetics of *Salmonella* genomic island 1. *Microbes Infect* **2006**, *8*, 1915-1922.
7. Gillings, M.R.; Gaze, W.H.; Pruden, A.; Smalla, K.; Tiedje, J.M.; Zhu, Y.G., Using the class 1 integron-integrase gene as a proxy for anthropogenic pollution. *ISME J* **2015**, *9*, 1269-1279.
